# Lipofibromatous Hamartoma of the Median Nerve: A Case Report

**DOI:** 10.7759/cureus.33516

**Published:** 2023-01-08

**Authors:** Ahmed M Elbayer, Sara Alharami, Ahmed H Elhessy

**Affiliations:** 1 Plastic and Reconstructive Surgery, The University of Tennessee Health Science Center, Memphis, USA; 2 Department of Plastic Surgery, Hamad General Hospital, Doha, QAT; 3 Orthopedics, The Rubin Institute for Advanced Orthopedics, Sinai Hospital, Baltimore, USA

**Keywords:** median nerve entrapment, lipofibroma, lfh, median nerve, lipofibromatous hamartoma

## Abstract

Lipofibromatous hamartoma (LFH) is a rare benign peripheral nerve tumor. The median nerve (MN) is most commonly affected in the upper extremity. We report a case of a 39-year-old male with LFH of the median nerve presented with swelling and symptoms of carpal tunnel syndrome treated successfully with decompression. LFH is reported with various descriptions because of the proliferative nature of its adipocytes and the fibrofatty infiltration within the peripheral nerves. Swelling around the volar aspect of the wrist remains the most frequent presentation of LFH. Surgical decompression without tumor resection can result in symptom improvement. In addition, post-decompression nerve coverage can be a solution to improve the residual hyperesthesia symptoms.

## Introduction

Lipofibromatous hamartoma (LFH) is a rare benign peripheral nerve tumor. LFH can be found in various peripheral nerves, but the median nerve is most commonly affected in the upper extremity. LFH is characterized by slow progression due to the proliferation of mature adipose cells within the epineurium and perineurium of a peripheral nerve [1-6]. LFH of the median nerve can be asymptomatic or present with pain, swelling, numbness, paraesthesia, or carpal tunnel syndrome [1,4,7,8].

We report a case of LFH of the median nerve presented with swelling and symptoms of secondary carpal tunnel syndrome treated successfully with decompression.

## Case presentation

**Figure 1:**
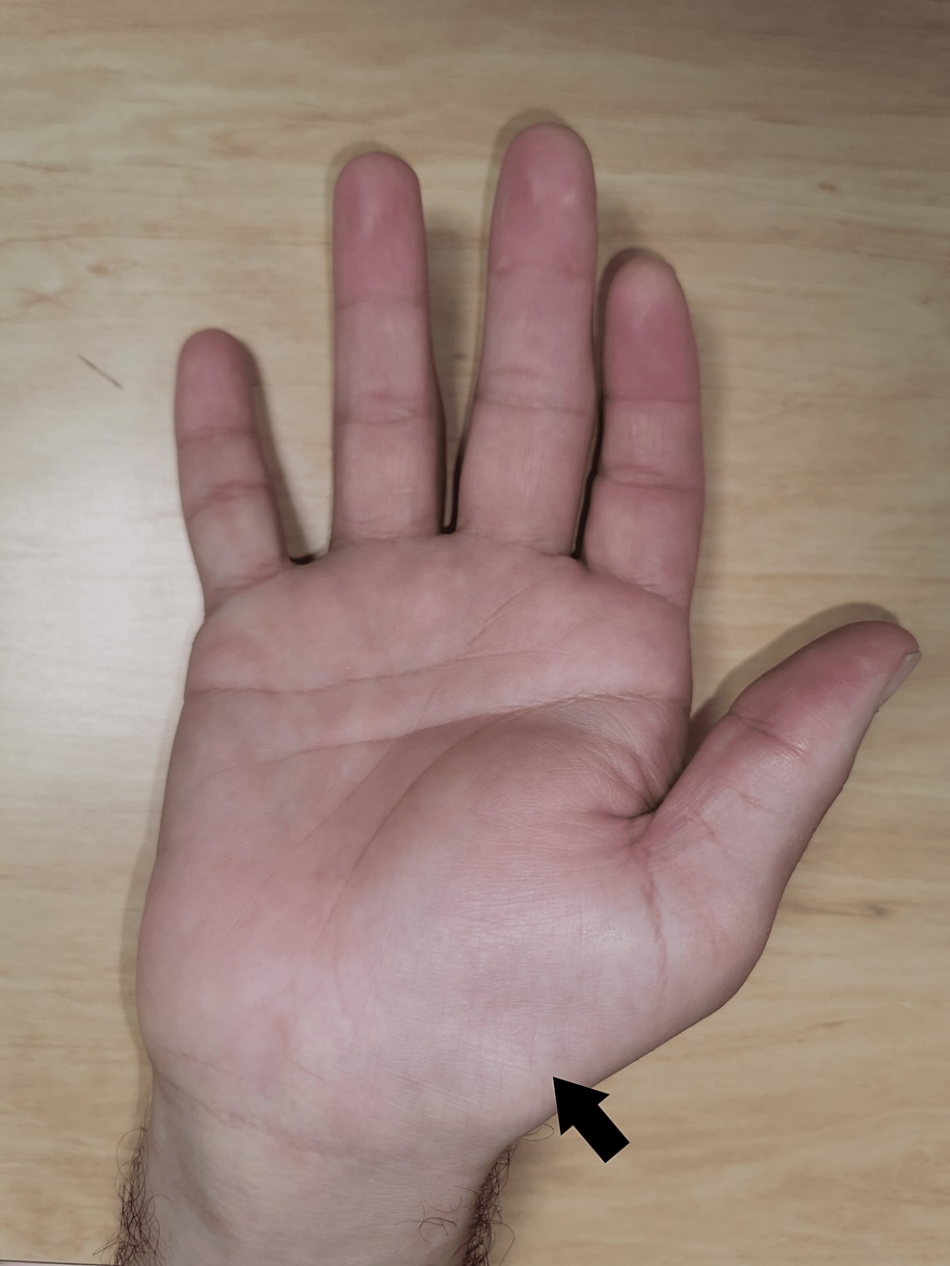
Clinical photograph showing the volar aspect of the right hand with a swelling proximal to the wrist crease and wasting of the thenar eminence (arrow)

A 39-year-old, right-hand dominant male presented to our clinic with a 10-year history of dysesthesia and paraesthesia affecting his right thumb, index, and long finger. Over the past 12 months, he noticed a wrist swelling with more persistent symptoms and started to experience some weakness in his grip strength. A physical examination of his right-hand revealed wasting of the thenar eminence and a tender swelling proximal to the wrist crease (Figure 1). Alteration of sensation along the median nerve distribution in the right hand was detected using the Semmes-Weinstein monofilament test.

Tinel sign was positive, and Phalen's maneuver was painful to perform. Electrodiagnostic studies (nerve conduction studies [NCS] and electromyography [EMG]) of the median nerve showed prolonged sensory and motor latency. The patient refused further radiological investigations and opted to proceed with surgical exploration and decompression.

**Figure 2:**
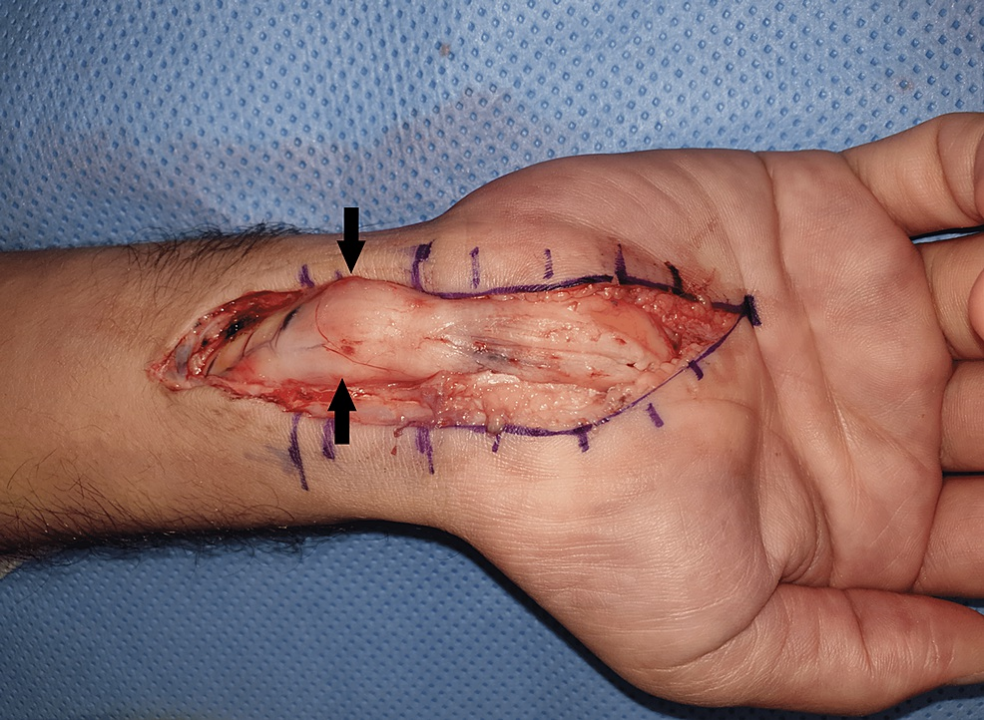
Intraoperative clinical photo showing the LFH tumor (arrows) and inflammation, edema, and perineural thickening with fibrosis of the median nerve LFH - lipofibromatous hamartoma

Under general anesthesia, a longitudinal lazy S-shaped incision was made at the wrist. After the transverse carpal ligament division, the dissection was extended proximally through the forearm fascia and revealed a 12.5x9.7 mm fusiform mass representing the LFH tumor. Inflammation, edema, and perineural thickening with fibrosis are noticed in the affected median nerve portion (Figure 2).

External neurolysis, involving freeing the median nerve from adhesions, was completed. Then, a nerve biopsy was taken for histopathology, which confirmed the diagnosis of LFH. The surgical wound was closed in a regular fashion.

**Figure 3:**
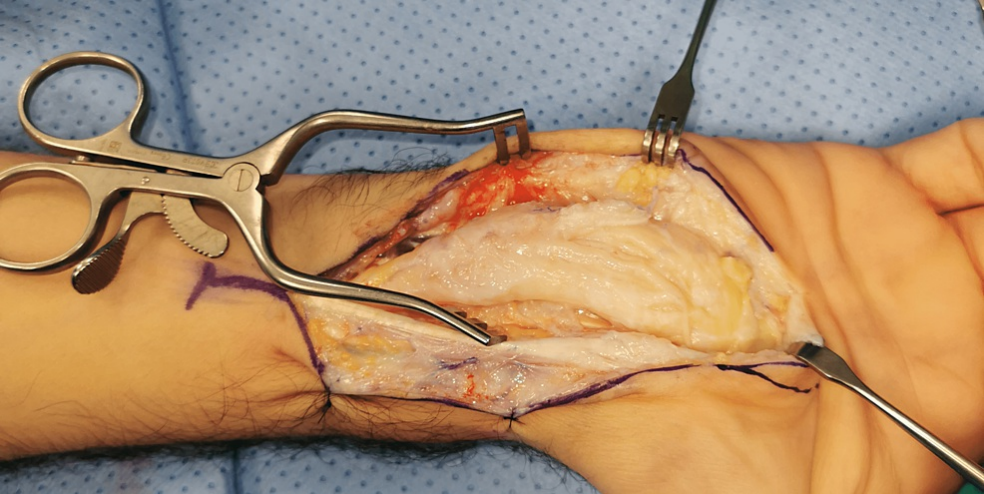
Intraoperative clinical photo of the median nerve during the second surgery showing resolution of the signs of nerve inflammation after the decompression surgery

The patient's hand was kept in a removable splint for two weeks, and he had three sessions with occupational therapy (OT) for education on suture site care, range of motion, finger mobility, heavy lifting, and scar mobility. The postoperative outpatient visits were scheduled after one week, two weeks, four weeks, six weeks, three months, six months, and one year. During the postoperative period, there was a gradual improvement in numbness. The patient noted hyperesthesia related to touch, and examination showed positive Tinnel's sign over the tumor site. Five months later, median nerve exploration was performed, which showed the resolution of the previous signs of nerve irritation (Figure 3).

**Figure 4:**
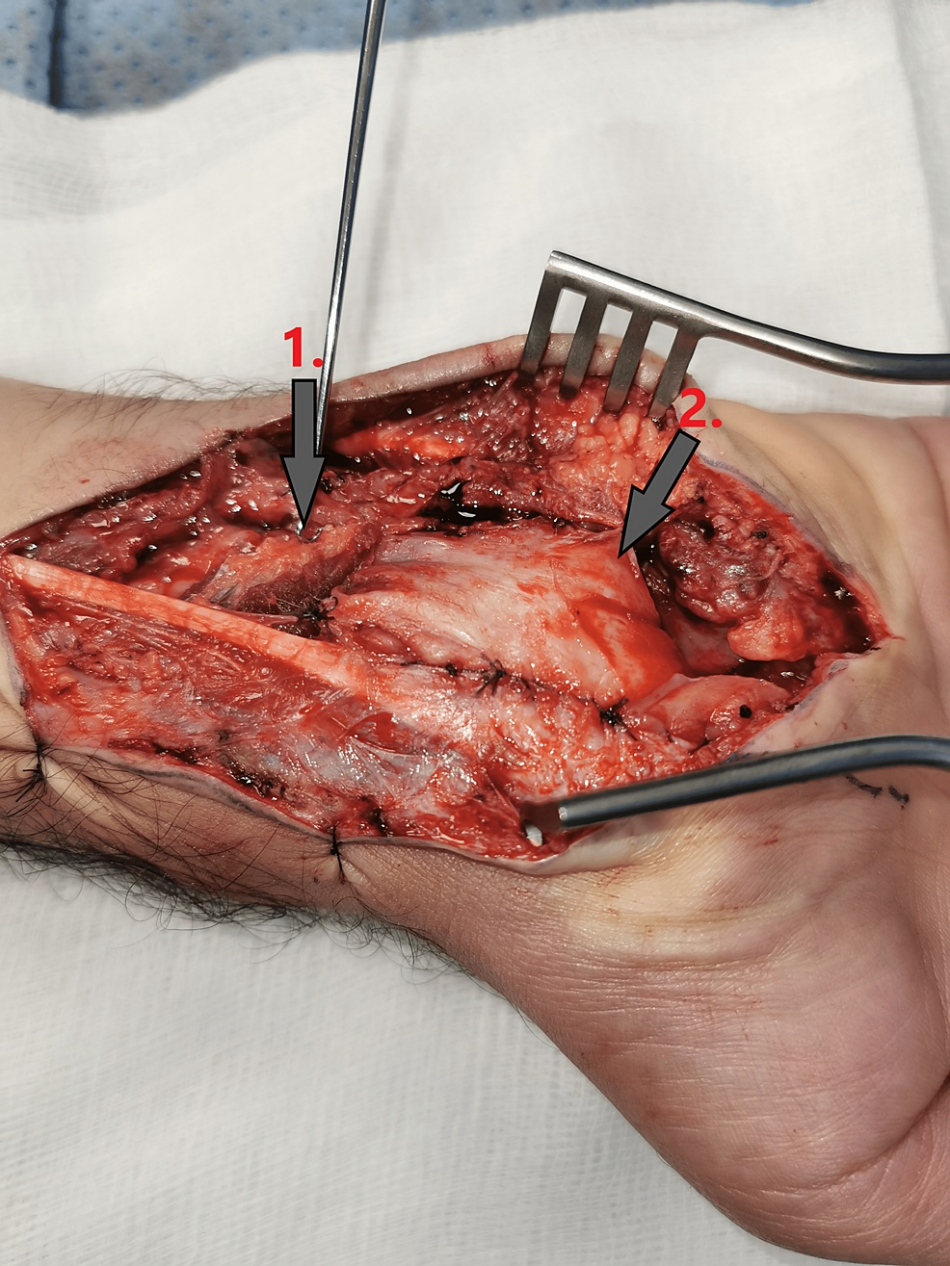
Intraoperative clinical photo showing pronator quadratus muscle local flap (arrow 1) and skin substitute (arrow 2) for median nerve coverage

Accordingly, another procedure was performed for proper nerve coverage (Figure 4). A pronator quadratus muscle local flap and skin substitute for nerve coverage used an acellular dermal substitute MatriDerm® (MedSkin Solutions, Billerbeck, Germany). 

The patient's paresthesia improved by four weeks, and he could resume his daily activities without limitations. Paresthesia resolution continued to 95% at the six-month follow-up. No improvement in the thenar muscle atrophy was noted after surgery, but the grip and pinch strength started to show clinical improvement (six months after surgery) compared to the preoperative assessment. Finally, the patient completed a two-year follow-up period with a complete resolution of his sensory symptoms. 

## Discussion

Lipofibromatous hamartoma is a rare fibro-fatty tumor that affects peripheral nerves. It has been reported in the literature with various descriptions, such as lipofibroma, intraneural lipoma, fatty infiltration of the median nerve, or lipomatous hamartoma [9]. Variation in LFH description comes from the nature of the tumor, which is characterized by the proliferation of the mature adipocytes leading to fibrofatty infiltration within the peripheral nerves epineurium and perineurium [1-7,9,10]. Between chronic nerve irritation, abnormal development of the flexor retinaculum, and antecedent trauma, the etiology of LFH remains unclear [11-15]. LFH has an early presentation with macrodactyly during childhood in 30% of the cases [10,14,16]. Swelling around the volar aspect of the wrist remains the most frequent presentation of LFH. The swelling usually appears several years prior to the onset of neurological symptoms [10]. An intraoperative biopsy is essential to rule out other malignant lesions, including neurofibrosarcomas [10,17].

Signs of chronic nerve compression are usually related to alteration in the blood-nerve barrier, followed by histological changes in the myelinated fibers. Morphologic and functional changes usually follow this process if decompression is not performed [18-20]. In our case, chronic compression was related to the long history of symptoms, followed by the recent increases in the symptoms' severity and thenar eminence wasting. Intraoperatively, the median nerve was inflamed, edematous, and thickened. Although surgical excision of LFH is not recommended for several reasons, prophylactic decompression is considered even in the absence of neurological symptoms [7,10,12,15]. In our case, the patient's neurological symptoms resolved after the decompression surgery. Due to persistent hyperesthesia, another procedure was done five months after decompression for proper nerve padding and coverage. After completing a two-year follow-up, the patient remained asymptomatic.

Finally, lipofibromatous hamartoma of the median nerve is a rare peripheral nerve tumor. Proper recognition starts with proper assessment, including the patient's history and clinical examination. The radiological evaluation can involve ultrasound and magnetic resonance imaging, but intraoperative biopsy is essential to confirm the diagnosis and to rule out other malignant conditions. Surgical decompression without tumor resection can result in symptom improvement. In addition, post-decompression nerve padding or coverage can sometimes improve residual hyperesthesia symptoms.

## Conclusions

Lipofibromatous hamartoma of the median nerve is one of the peripheral nerve's rare, slowly growing, and benign tumors. Early decompression is preferred, and intraoperative biopsy is valuable. Together with nerve decompression surgery, surgeons should consider proper median nerve coverage in selected cases to avoid post-operative hyperesthesia.
